# Intraventricular Topotecan for the Treatment of Neoplastic Meningitis: Five Case Studies

**DOI:** 10.6004/jadpro.2012.3.4.4

**Published:** 2012-07-01

**Authors:** Julie Walker, Diana Schultz, Kathleen Grisdale, Morris Groves

**Affiliations:** From The University of Texas MD Anderson Cancer Center, Houston, Texas

## Abstract

Many systemic cytotoxic agents cannot penetrate the blood-brain barrier. Because of this, in approximately 5% of cancer cases, metastatic disease is seen in the leptomeninges. Without treatment, patients with neoplastic meningitis (NM) generally survive for only a matter of weeks. In the treatment of NM, intraventricular (IVT) topotecan results in progression-free and overall survival outcomes similar to those seen with other IVT chemotherapies, while being particularly well tolerated by most patients. In this article, we present the case studies of five patients with NM, with various primary malignancies, who received treatment with IVT topotecan.

As patients experience longer survival times with chronic malignancies, the leptomeninges (arachnoid and pia mater) may increasingly play host to metastatic disease from a variety of primary cancers. This occurs in approximately 5% of cancer cases (Chamberlain, 2008); due to the inability of many systemic cytotoxic agents to penetrate the blood-brain barrier, the leptomeninges can become a sanctuary for a number of metastatic malignancies (Groves et al., 2008). Without treatment, patients with neoplastic meningitis (NM) have an overall survival of only a few weeks (Groves, 2010).

While some authorities argue that treatment is only palliative, it provides an important means of stabilizing the patient and preventing further neurologic decline (Glantz, Van Horn, Fisher, & Chamberlain, 2010). Intrathecal (IT) and intraventricular (IVT) chemotherapy via an implanted ventricular reservoir circumvents the blood-brain barrier and blood–cerebrospinal fluid (CSF) barrier to enable the drug to have direct contact with the tumor cells. However, the chemotherapeutic agents commonly used to treat NM via the IVT or IT route often cause intolerable arachnoiditis evidenced by headache, nausea, and vomiting (Beauchesne, 2010). If untreated, arachnoiditis may cause life-threatening increased intracranial pressure. Toxicities of IT chemotherapy often result in dose reductions, increased use of steroids, and/or discontinuation of the chemotherapy. Research into effective and well-tolerated treatments continues (Groves, 2010). The use of IVT topotecan in the treatment of NM results in progression-free and overall survival outcomes similar to those of other IVT chemotherapies, while being particularly well tolerated by most patients (Groves et al., 2008).

Presented here are case studies from five patients who received IVT topotecan for NM. Primary tumors were as follows: two patients with primary breast cancer, one patient with multiple myeloma, one patient with medulloblastoma, and one patient with melanoma. Overall, we found that IVT topotecan was well tolerated with few symptoms of arachnoiditis, making it a viable treatment for patients with NM originating from a variety of primary tumor sites.

## Case Study 1: Multiple Myeloma

A 56-year-old man who was diagnosed with a primary cancer of multiple myeloma in January 2006 presented with lethargy, headache, weakness, and seizures. On August 6, 2007, magnetic resonance imaging (MRI) of the brain revealed abnormal enhancement of the genu/body of the corpus callosum, more prominent on the right; intensive leptomeningeal enhancement involving the basal cisterns, medial cortical sulci, brainstem, vermis, and bilateral auditory canals; and mild hydrocephalus. Whole-spine MRI on August 14 revealed extensive intrathecal disease in the cervical and thoracic spine, conus, and cauda equina. Subsequent lumbar puncture revealed typical immature plasmacytoid cells consistent with involvement by multiple myeloma.

On August 10, 2007, the patient underwent placement of an Ommaya reservoir. He then underwent whole-brain radiation to a total of 30 Gy, which was completed on August 20. Following this, radiation therapy was delivered to the whole spine, consisting of 25 Gy over 10 fractions at 2.5 Gy per fraction to the cervical, thoracic, and lumbar spine. On September 27, treatment with IVT topotecan was started. Other treatments during IVT topotecan therapy included donor lymphocytes, bortezomib (Velcade), dexamethasone, cyclosporine, epoetin alfa, and radiation to a pelvic mass.

The patient experienced arachnoiditis symptoms while on topotecan, including gait ataxia, confusion, and slurred speech, but he received no special interventions. Over the course of treatment, serial brain MRI revealed white matter changes consistent with mild toxicity.

The total length of the patient’s treatment with IVT topotecan was 5 months; it was then discontinued due to progression of primary disease. His Karnofsky performance status (KPS) scores at the beginning and end of treatment for NM were 70 and 60, respectively.

## Case Study 2: Breast Cancer

In 2007, a 32-year-old woman was diagnosed with invasive carcinoma of the left breast (estrogen-receptor positive, progesterone-receptor negative, HER2/*neu* negative), with metastasis to the lung and bone at the time of diagnosis. In January 2009, the patient was diagnosed with NM in the lumbosacral spinal cord as well as in the cerebellum. Symptoms at the time of NM diagnosis included decreased mobility, paresthesia and weakness in the right lower extremity, and constipation. Objective findings at the time of NM diagnosis included right foot weakness, sensory loss, saddle paresthesia, MRI enhancement of the cerebellum, bilateral hemispheres, folia cerebella, and epidural enhancement of S1-2 in the sacral spine. Cerebrospinal fluid cytology was persistently negative.

The patient underwent whole-brain radiation at 35 Gy in 14 fractions followed by radiation to the sacral spine; the total radiation dose was 36 Gy in 12 fractions. She had some improvement in right lower extremity symptoms following radiation. On April 20, 2009, she underwent placement of an IVT reservoir; on April 30, she began IVT topotecan. Other treatments received during treatment with topotecan included capecitabine (Xeloda), vinorelbine, nab-paclitaxel (Abraxane), zoledronic acid, paclitaxel, and spinal radiation.

The patient experienced arachnoiditis symptoms during treatment with IVT topotecan, including headache, fever, chills, and stiff neck. She received no special intervention for any of these symptoms. Serial MRI brain imaging over the course of the patient’s treatment with IVT topotecan revealed no evidence of toxicity.

The patient’s total length of treatment with IVT topotecan was 9 months; treatment was discontinued due to progression of her primary cancer. Her KPS scores at the beginning and end of her treatment with IVT topotecan were 90 and 20, respectively.

## Case Study 3: Melanoma

**Figure 1:**
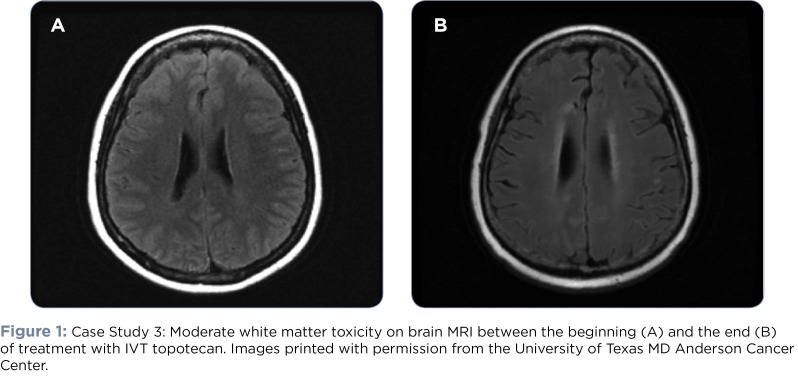
Figure 1: Case Study 3: Moderate white matter toxicity on brain MRI between the beginning (A) and the end (B)of treatment with IVT topotecan. Images printed with permission from the University of Texas MD Anderson Cancer Center.

A 54-year-old woman was initially diagnosed with melanoma of the left posterior shoulder in 2002. In 2007, she was found to have widely metastatic disease involving the lung, liver, gastrointestinal tract, and left parietal lobe of the brain. On January 22, 2009, a brain MRI was suspicious for NM (see Figure 1). Subsequently, an IVT reservoir was placed on February 17. At the time of reservoir placement, the CSF was positive for malignant cells consistent with melanoma.

Symptoms at the time of NM diagnosis were positive for only residual paresthesias noted with original parietal metastasis. Objective findings at the time of diagnosis included ependymal enhancement around the left frontal horn and posterior aspect of the left occipital horn consistent with CSF dissemination of disease. The patient had no specific treatment for NM prior to the initiation of IVT topotecan. She had no treatments concurrent with IVT topotecan. Symptoms during the time of IVT treatment that could have been related to arachnoiditis included nausea and headache. She received dexamethasone as an intervention and had good results. Serial MRI imaging over the course of treatment revealed moderate evidence of toxicity.

The patient was treated with IVT topotecan for a total of 9 months; treatment was discontinued due to radiographic progression of NM. Her KPS scores at diagnosis and at the end of topotecan treatment were 90 and 20, respectively.

## Case Study 4: Medulloblastoma

In 2001, a 37-year-old man received a primary diagnosis of medulloblastoma after complaining of diplopia. In July 2008, he was diagnosed with NM. Subjective symptoms at the time of NM diagnosis included fatigue, numbness along the left edge of his tongue and along the left cheek and jaw area, and hypogeusia. Objective findings included T2 hyperintensity and enhancement seen overlying the cerebellar vermis. A lumbar puncture was performed, revealing malignant cells within the CSF consistent with a diagnosis of medulloblastoma. On July 21, 2008, the patient had an IVT reservoir placed and surgical removal of his recurrent medulloblastoma.

On August 1, the patient received his first IVT treatment with topotecan. Treatments administered concurrently with IVT topotecan included systemic treatment with etoposide, cisplatin, and cyclophosphamide. Arachnoiditis symptoms included fatigue, nausea, vomiting, neck stiffness, lethargy, seizures, visual hallucinations, and headaches, for which dexamethasone was given.

The patient’s total length of treatment was 12 months. Serial brain MRI over the course of treatment revealed no evidence of toxicity. Treatment was stopped at the patient’s request due to progression of NM. His KPS scores at the beginning and end of his treatment for NM were 80 and 90, respectively.

## Case Study 5: Breast Cancer

In 2005, a 43-year-old woman was diagnosed with cancer of the left breast. She was found to have metastasis to the lung in 2007 and then brain metastasis and leptomeningeal enhancement of the cerebellum, vermis, and sylvian fissure in April 2009. She underwent whole-brain radiation followed by placement of an IVT reservoir in June 2009.

The patient’s subjective symptoms at diagnosis of NM included headache, constipation, unilateral hearing loss, and paresthesia. Objective findings at the time of NM diagnosis included right hemibody weakness, gait instability, urinary retention, radiographic evidence of metastasis to leptomeninges of the cerebellum extending into sylvian fissures, mass effect compressing left optic chiasm, leptomeningeal enhancement of T2-3, 7 and intrathecal roots in the lumbar sac. Neoplastic meningitis treatment prior to receiving IVT topotecan consisted of whole-brain radiation. Other treatments given concurrently with IVT topotecan included capecitabine, spinal radiation, cisplatin, and lapatinib (Tykerb). Symptoms of arachnoiditis during IVT topotecan treatment included headache, vomiting, and delirium; however, no interventions were documented. Serial MRI brain images over the course of treatment with IVT topotecan revealed mild evidence of toxicity.

The patient was treated with IVT topotecan for a total of 5 months. It was then discontinued due to radiographic progression of NM. Her KPS scores were 80 and 10 at the beginning and end of treatment with topotecan, respectively.

## Results

In five patients receiving IVT topotecan, the average KPS at diagnosis of NM was 82. The average length of treatment was 8 months. All five patients had symptoms of arachnoiditis while receiving IVT topotecan, but only two patients had symptoms severe enough to require intervention. Further, it is unclear whether symptoms were related to topotecan, disease, or other concurrent treatments. The average KPS at the end of treatment was 40. Radiographic evidence of white matter toxicity occurred in three patients, but the etiology was difficult to determine as each had also received whole-brain radiation. See Table 1 for functional status and toxicity information for all five patients.

**Table 1:**
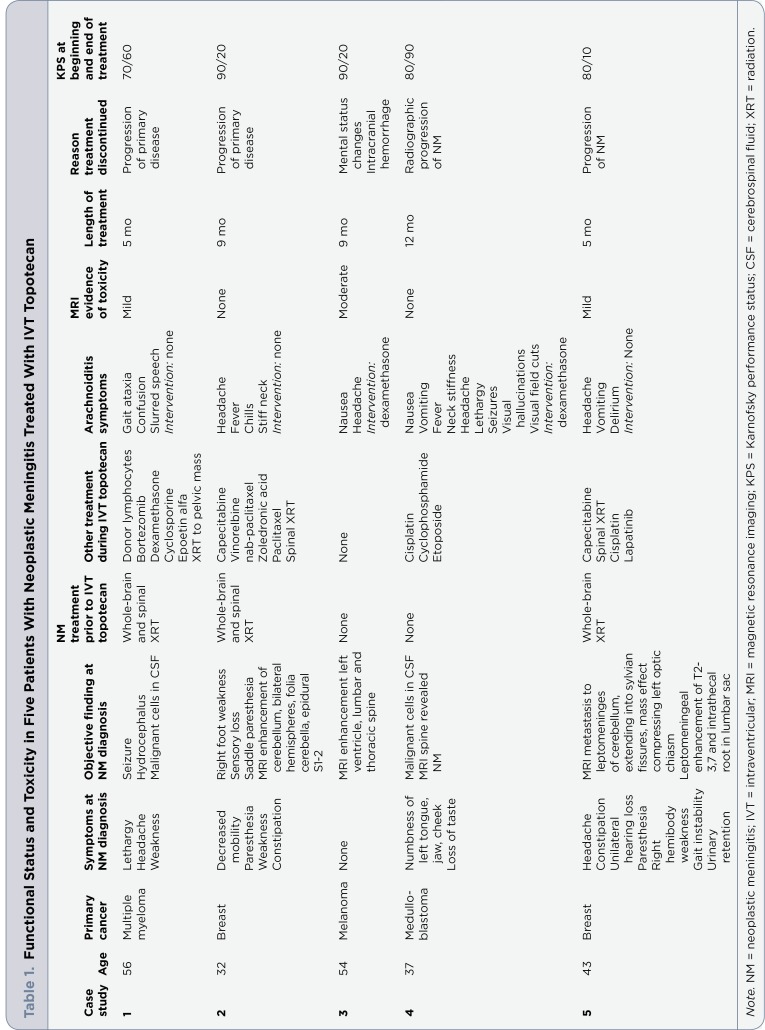
Functional Status and Toxicity in Five Patients With Neoplastic Meningitis Treated With IVT Topotecan

## Conclusions

Even though all patients experienced nausea, vomiting, headache, or other neurologic symptoms while receiving IVT topotecan, symptoms were not generally severe enough to limit treatment. Dexamethasone was documented as the only treatment intervention. The average duration of treatment was 8 months, which may correspond to the study by Groves et al. reporting a 19% 6-month progression-free survival, with 32% of those patients reporting mild arachnoiditis (Groves et al., 2008).

Unfortunately, there are many confounding factors when trying to determine the cause of neurologic symptoms in NM patients receiving IVT topotecan. Imaging studies may reveal treatment-related toxicities. However, due to its limited toxicity and its equivalence in efficacy outcomes when compared with other IVT chemotherapies, IVT topotecan should be considered a front-line choice for IVT chemotherapy in those patients for whom this local-therapy approach is deemed appropriate (Groves et al., 2008). Further, IVT topotecan warrants further investigation in combination with other IVT chemotherapies and in combination with newer systemic chemotherapies. Well-designed, randomized studies testing the efficacy and tolerability of IVT topotecan, including a quality-of-life component, are needed.

Advanced practitioners have the opportunity to play a crucial role in the detection of subtle neurologic changes that may signal initial metastatic involvement of the leptomeninges or adverse reactions to treatment. Responsibilities of the advanced practitioner include symptom management and ordering appropriate consultations and services such as physical therapy, supportive care, and home health and home safety evaluations. Advanced practitioners should play an active role in the design and implementation of research studies related to NM, particularly in advocating for the inclusion of measurable quality-of-life components.
